# ‘Making Moves’: Protocol for a hybrid effectiveness-implementation pre-post trial of a co-designed online physical activity program for childhood cancer survivors

**DOI:** 10.1016/j.jsampl.2025.100099

**Published:** 2025-04-12

**Authors:** Lauren Ha, Claire E. Wakefield, Jacqueline Jacovou, Karen Johnston, Donna Drew, Mark W. Donoghoe, David Mizrahi, Richard De Abreu Lourenco, Richard J. Cohn, Natalie Taylor, Christina Signorelli, Youth Advisory Board, Kiarn Roughley, Callum Joyner, Aj Wright

**Affiliations:** a Behavioural Sciences Unit, Discipline of Paediatrics & Child Health, School of Clinical Medicine, Randwick Clinical Campus, UNSW Medicine & Health, UNSW SYDNEY, Australia; b Kids Cancer Centre, Sydney Children’s Hospital, Randwick, NSW, Australia; c Clinical Research Unit, UNSW Medicine & Health, UNSW, Sydney, Australia; d The Daffodil Centre, The University of Sydney, a Joint Venture with Cancer Council NSW, Australia; e Centre for Health Economics Research and Evaluation, UTS, Sydney, Australia; f School of Population Health, UNSW Medicine & Health, UNSW, Sydney, Australia

**Keywords:** Childhood cancer, Survivorship, Physical activity, Exercise, Digital health, Implementation

## Abstract

**Background:**

Childhood cancer survivors are at increased risk of developing chronic health conditions, including cardiovascular disease. Cardiovascular disease risk is further exacerbated by low physical activity levels and high levels of sedentary behaviour. Yet many survivors do not meet the physical activity guidelines, and those living in regional and remote areas have limited access to exercise interventions and cancer care programs. Therefore, it is critical that physical activity programs are accessible for childhood cancer survivors, no matter where they live. This protocol describes the development and planned evaluation of ‘Making Moves’, a co-designed online physical activity program for childhood cancer survivors aged 8–21 years.

**Methods and analysis:**

This type I hybrid effectiveness-implementation pre-post trial will simultaneously (i) test the potential effectiveness of Making Moves on survivors' physical activity self-efficacy, and (ii) explore barriers and facilitators to implementation from multiple levels (individual, provider, organisational, and community) to inform future adaptations and implementation strategies. Making Moves includes an 8-week online program offering eight age-appropriate health behaviour educational modules with guided home-based physical activity videos, and up to five tailored telehealth sessions with an Accredited Exercise Physiologist. Assessment of the primary outcome (physical activity self-efficacy, i.e., perceived ability to engage in physical activity) and secondary outcomes (physical activity levels, aerobic fitness, muscular strength, symptoms of depression, readiness to exercise, perceived enjoyment of exercise, health-related quality of life) will occur at baseline (week 0), post-intervention (week 9), and follow-up (6 months). To assess the factors affecting the program’s implementation, we will conduct a process evaluation guided by the Consolidated Framework for Implementation Research 2.0 to interview survivors and parents, and future potential implementors. To judge the potential implementation success of Making Moves, we will collect implementation data (feasibility, acceptability, costs) for our process evaluation throughout the trial.

**Ethics and dissemination:**

Ethical approval was obtained from the Sydney Children’s Hospital Network Human Research Ethics Committee (2023/ETH01614). We will publish our findings in peer-reviewed journals, present findings at relevant medical and scientific conferences, and disseminate research updates via newsletters to stakeholders and community networks.

**Trial registration number:**

ANZCTR12623000188639. Registered 22 Feb 2023.

## 1 Background

The cancer survivor population is rapidly growing due to treatment advances and improvements in supportive care [1]. In Australia alone, approximately 800 children and adolescents aged up to 14 years are diagnosed with cancer each year [2] and more than 200,000 globally [3]. With survival rates increasing to >85 ​% in high-income earning countries such as Australia [4], the long-term needs of patients surviving childhood cancer is changing. The prevalence of developing at least one chronic health condition post childhood cancer treatment is estimated to be as high as 93 ​%, 35 years after cancer diagnosis [5]. Compared to their siblings, survivors of childhood cancer have a significantly greater risk of developing long-term health complications, such as heart disease and obesity, arising decades beyond treatment completion [6].

Evidence for the benefits of physical activity in this population is increasing, including improvements in symptom management (e.g., pain and fatigue), physical and psychosocial wellbeing (e.g., improving body composition and mood), and extending the length of survivorship [[7], [8], [9]]. Yet many young survivors engage in consistently low levels of physical activity and have high levels of sedentary behaviour [10]. Among adult survivors of childhood cancer, many report decreasing activity levels throughout adulthood, and 48 ​% report levels below the recommended guidelines [11]. Individuals with strong beliefs in their physical activity self-efficacy are more likely to be physically active and are able to adopt and maintain physical activity even if external barriers emerge [12]. Prior research demonstrates that physical activity self-efficacy (defined as one’s belief or confidence in their capability to engage in physical activity) is low among childhood cancer survivors [13]. To promote regular and long-term physical activity behaviours among childhood cancer survivors, there is a critical need for lifestyle interventions to include components that target physical activity self-efficacy.

Many survivors face multiple barriers to physical activity participation such as physical barriers (e.g., fatigue), motivational barriers (e.g., preference to do something else), and logistical barriers (e.g., lack of time) [14]. One challenge of engaging in physical activity in this population is the limited access to health behaviour programs across Australia. Families living in regional and remote areas are also burdened by distance and long travel to access supportive care [15], such as services provided by Accredited Exercise Physiologists (AEPs) to promote, prescribe, and deliver physical activity. As a result, regional/rural families have less access to supportive care, experience higher cancer-related financial hardship, and are more likely to experience adverse health outcomes [16,17]. To overcome the barrier of distance and travel, distance-delivered interventions are promising solutions that can improve accessibility and promote health behaviours [18,19]. Yet there remains a gap in understanding the potential for digital health physical activity programs to be implemented within existing healthcare or community systems [20]. Few studies have examined the factors that are crucial to the implementation of digital health physical activity programs, to maximise their uptake and sustainability in a real-world setting. Therefore, we aim to:

(i) Evaluate the potential effectiveness of Making Moves on childhood cancer survivors' physical activity self-efficacy and other health outcomes (e.g., physical activity levels, aerobic fitness levels, muscular strength) at baseline (week 0), post-intervention (week 9, our primary endpoint), and follow-up (6 months).
(ii) Explore barriers and facilitators to implementation at multiple levels (i.e., individual, provider, organisational and community) to inform future adaptations and implementation strategies.

## 2 Methods

We used the StaRI checklist [21] to report on the details of the intervention in conjunction with the SPIRIT 2013 checklist [22] to report on items for a clinical trial protocol.

### 2.1 Aims

The primary aim of this study is to examine the potential effectiveness of ‘Making Moves’, an online education program combined with tailored exercise support, on childhood cancer survivors' physical activity self-efficacy. The secondary aims include: to evaluate the potential effectiveness of Making Moves on survivors' physical activity levels, aerobic fitness levels, muscular strength, mental wellbeing, readiness to exercise, perceived enjoyment of exercise, and health-related quality of life. The study will also explore the barriers and facilitators to implementation of Making Moves from multiple levels (i.e., individual, provider, organisational and community) to inform future adaptations and implementation strategies. These multi-perspective data will help the future selection of implementation strategies to adapt and scale the Making Moves program. We will additionally collect intervention feasibility (i.e., recruitment rate, retention rate, participation rates and adverse events), acceptability (i.e., engagement, satisfaction) data, and evaluate the costs of the intervention and its implementation.

### 2.2 Study design

We will use a type I hybrid effectiveness-implementation single pre-post trial to evaluate the potential effectiveness and implementation of Making Moves [23]. The traditional process from intervention development to implementation is estimated to take an average of 17 years [24]. Hybrid effectiveness-implementation study designs offer a potential solution to this delay by accelerating the process by simultaneously investigating both the effectiveness of the intervention and its implementation [25]. Therefore, we will evaluate the effectiveness outcomes of our intervention whilst exploring ways to support implementation in the real world. Survivors who opt in will participate in both the effectiveness (online intervention) and implementation (semi-structured interview) elements of the study. We will also interview potential future implementors of Making Moves, including healthcare professionals (HCPs) and representatives from community-based organisations to understand what implementation strategies they may need to implement the program and reach this population, or how Making Moves might need to be adapted, whilst retaining its core functional components to optimise implementation [26].

### 2.3 Prior work

We have already established the feasibility and acceptability of our online education program among childhood cancer survivors in our pilot study (formally known as ‘iBounce’) [27]. Feedback from this pilot study highlighted that survivors were satisfied with the overall program and 86 ​% reported having learned from the program. Additionally, survivors' aerobic fitness levels significantly increased after completing the intervention. However, some parents were dissatisfied due to technical difficulties and many survivors preferred a different activity tracker. To address the technical issues, we updated the platform (app) to a website that is compatible with internet-enabled devices (laptop, tablet, or smartphone) and modified the type of activity tracker, removing the need to synchronise step count data to the online program. Subsequently, we conducted multi-perspective focus groups and interviews with childhood cancer survivors, parents, and HCPs to explore their experiences and priorities of using digital health interventions to engage survivors in physical activity. We used the self-determination theory to guide our intervention development and evaluation [28]. The self-determination theory predicts that users with intrinsic motivation to engage in a health behaviour will be enhanced by users' autonomy (i.e., feeling self-directed), competence (control and confidence), and perceived relatedness (support from the intervention). We used these three constructs as our intervention design objectives to inform key intervention features that were prioritised by survivors, parents, and HCPs from our focus groups (Table 1). One key intervention feature was to include video formatting to engage survivors. Exercise videos were created and filmed by exercise physiologists in training and range between 1 and 20 ​min, focusing on aerobic fitness, muscular strength and endurance, flexibility, and motor skill development. All videos have been approved by an AEP and are deemed safe for participants to follow along to at home. Using our in-depth qualitative research combined with ongoing collaborative meetings with our Youth Advisory Board (three consumers involved in the conception and development of this study and design of the program), we modified iBounce and updated the intervention for childhood cancer survivors aged 8–21 years, known as ‘Making Moves’.

**Table 1:** Guiding principles to intervention development as defined by our Youth Advisory Board.

Intervention design objectives	Key intervention features
To promote user autonomy	• Survivors have the choice to engage in aspects of the intervention including creating their own goals (with guidance from an HCP), viewing health behaviour education, and choosing physical activity videos to follow along to at home. • Survivors have the choice as to how they engage with the intervention such as the timing.
To promote user competence	• Our educational modules provide clear structure and an optional check-in telehealth session during weeks 2–3. • In collaboration with our Youth Advisory Board, we co-designed the program content including a blog post written by a consumer about their story of overcoming barriers and returning to physical activity. • Making Moves incorporates graded goalsetting that is led by the AEP in telehealth sessions. Goals are created in partnership between survivors and the AEP. • Survivors receive tailored feedback based on their goal achievements during their telehealth sessions with the AEP.
To promote a sense of relatedness	• The making Moves educational modules are written in autonomy-supportive language, provide commonly asked questions and address common concerns identified by our Youth Advisory Board. • The educational modules provide bite-sized information displayed across different mediums (i.e., text, video, interactive text, images) to ensure the information is interesting and engaging for the user. • The making Moves program interface follows best practice for maximising usability to ensure users can navigate the program.

Abbreviation: AEP: Accredited Exercise Physiologist.

### 2.4 Participant eligibility and recruitment

**Fig. 1:**
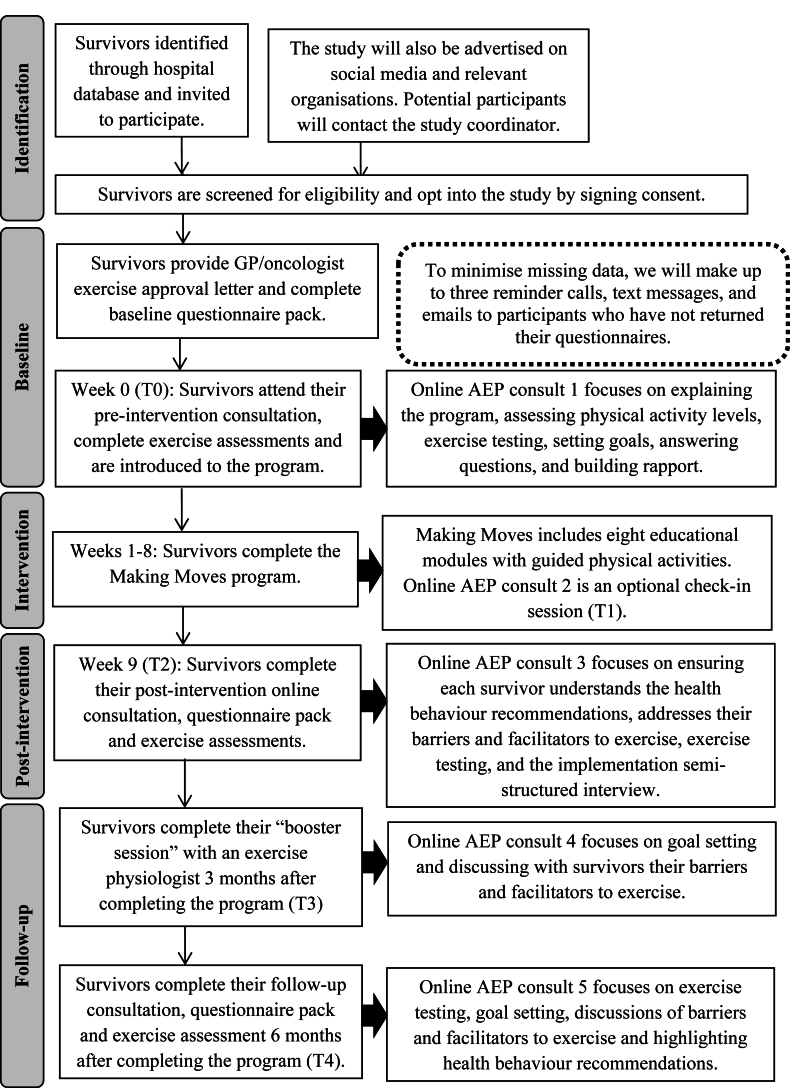
Study design diagram. Abbreviations: AEP: accredited exercise physiologist; GP: general practitioner; T0: baseline, T1: week 2–3; T2: post-intervention (week 9); T3: 3-months post-intervention; T4: 6-months post-intervention. Online AEP consult 5 focuses on exercise testing, goal setting, discussions of barriers and facilitators to exercise and highlighting health behaviour recommendations.

Study inclusion criteria include any individual in Australia who i) was diagnosed with cancer before the age of 18 years, ii) is aged 8–21 years at time of study participation, and iii) has completed cancer treatment at least six months prior or is receiving maintenance therapy. Exclusion criteria include any individual who i) is unable to provide informed consent, ii) is currently pregnant, iii) has not provided a medical approval letter to undertake unsupervised exercise or physical activity, iv) does not have internet access at home or webcam software, and v) is already participating in another research study that will affect this study’s primary or secondary outcomes. Clinical nurse consultants will identify potentially eligible patients treated at Sydney Children’s Hospital using clinic lists (see Fig. 1). A study team member will contact participants via phone to discuss the study and confirm eligibility. In addition, we will advertise nationwide via social media in the form of study posters, written posts, or study videos on platforms (e.g., Facebook), and relevant organisations and working groups (e.g., The Kids' Cancer Project). Interested participants will also be able to self-refer or be referred to the intervention by an HCP via an opt-in link on the Making Moves website. After receiving contact details from interested participants, a team member will contact participants via phone to confirm eligibility and discuss the study further. To opt in, eligible participants will return via email a signed consent form and exercise approval letter from their medical practitioner. Consent forms will be collected using Research Electronic Data Capture (REDCap).

For the implementation component, any individual who is a i) parent or caretaker of a young person diagnosed with cancer, ii) HCP, ii) representative from a community organisation relevant to health, young people, and/or oncology, or iv) member of the research team will be eligible to participate in semi-structured interviews. Potential HCPs and representatives from community-based organisations will be identified through investigators of this study or through ‘snowballing’ where we will approach participants and ask them to recommend any potential clinic staff or organisations who may be interested. A team member will contact potential participants via email or telephone with our study information pack to screen for eligibility. Other avenues of recruitment will be through social media posts, relevant working groups, committees, and newsletters. Details about the study will be provided in each social media post and study poster for interested participants to contact the study coordinator. Research staff who will be involved in the delivery of the intervention will also be invited to participate in semi-structured interviews to understand their perspectives on the factors likely to influence the implementation success of our intervention. HCPs and representatives from community organisations will be contacted throughout the effectiveness trial, whilst members of the research team will be invited at the end of the effectiveness trial.

### 2.5 Intervention

Making Moves is an 8-week online physical activity program that consists of age-appropriate health behaviour education, and guided physical activity videos, and is complemented by up to five telehealth exercise consultations with an Accredited Exercise Physiologist (AEP). Survivors who opt into the study will complete eight self-paced educational modules that focus on different physical activity and health behaviour topics, including healthy eating (Table 2). Each module contains educational content presented in text and short videos, exercise tips, and various guided exercise videos for survivors to follow along to at home. The intervention encourages survivors to follow the exercise videos, either individually or with a friend or family member, to learn new skills and increase their physical activity levels. Additionally, each exercise video contains modifications for the participant to make the activity easier or harder. To ensure the program is age-appropriate, the educational content readability and exercises have been modified slightly to address three age groups: i) 8–12 years, ii) 13–17 years, iii) 18–21 years. At the end of each module, survivors are encouraged to monitor their activity levels using an activity tracker. Survivors will be gifted with a Fitbit activity tracker to use as a motivational tool throughout the intervention period and beyond the study. If survivors already own an activity tracker, they will have the option to use their own instead. No data will be collected from the Fitbit activity tracker.

**Table 2:** Making Moves module topics and exercise video examples.

Module number	Module topic	Exercise video examples
1	Introduction to making Moves	• For 8–12 years: Warm up activities with friends, low intensity adventure game with friends • For 13–17 years: Warm up exercises, squat challenge with friends • For 18–21 years: Stretches you can do at your desk, jumping activities that focus on balance and coordination
2	Living a physically active lifestyle	• For 8–12 years: Basketball drills, running games • For 13–17 years: High intensity interval training, full body workout • For 18–21 years: Boxing drills, agility exercises
3	Sedentary behaviour	• For 8–12 years: Medley race with family/friends, exercises to get your heart rate up • For 13–17 years: Tabata exercise, cardiovascular exercise challenges with family/friends • For 18–21 years: Cardiovascular fitness shuttle run, functional mobility exercise
4	How much exercise do I need to do?	• For 8–12 years: Coordination exercises, ball exercises. • For 13–17 years: Squat variations, ‘luck of the draw’ heart pumping exercise routine • For 18–21 years: Strength exercises with explosive movements, warm up strength and stretch routine
5	Staying fit and motivated	• For 8–12 years: Adventure game with friends, plank exercise • For 13–17 years: Lower body exercise skills, 15-min interval workout with friends • For 18–21 years: Partnered abdominal workout, dynamic and static stretches
6	Muscle strength and endurance	• For 8–12 years: Wall sit challenge, strength game • For 13–17 years: Plank variations and challenges, strength circuit with varying levels of difficulty • For 18–21 years: Upper and lower body strength workout, introduction to common gym equipment.
7	Flexibility, balance, and coordination	• For 8–12 years: Ball coordination exercises with family/friends, tightrope walk game. • For 13–17 years: Soccer skills, yoga • For 18–21 years: Yoga, balance exercises with a partner
8	Healthy eating	• For 8–12 years: Balance game, ball catching drills with a partner • For 13–17 years: Coordination and control exercise, at home balance routine • For 18–21 years: Flexibility videos targeting different parts of the body, lower and upper body strength workout.

Survivors will also attend up to five telehealth exercise consultations with an AEP via Microsoft Teams at baseline (week 0; T0), week 2 or 3 (optional consultation; T1), post-intervention (week 9; T2), 3-month booster session (3-months post-intervention completion; T3), and follow-up (6-months post-intervention completion; T4). Each exercise consultation will focus on tailored physical activity coaching, exercise monitoring, goalsetting or reviewing goals, identifying any barriers or facilitators to exercise, and reinforcement of education from the online program. These consultations will also provide survivors the opportunity to ask questions to receive personalised feedback. The optional consultation at T2 is designed to troubleshoot any program-related problems and to reinforce program adherence. At T4, a summary of the last consult including their goals and recommendations made by the AEP will be emailed to survivors.

### 2.6 Outcome measures

#### 2.6.1 Data collection

Data will be collected from survivors via self-report questionnaires and AEP telehealth consultations. We will also collect data from parents, HCPs and community organisations via semi-structured interviews. Data including clinical and demographic information will be collected at T0 via REDCap.

#### 2.6.2 Primary outcome

##### 2.6.2.1 Physical activity self-efficacy

To assess the potential effectiveness of Making Moves, the primary outcome will be survivors' physical activity self-efficacy measured using the 8-item Physical Activity Self-Efficacy Scale (PASES) [29] at T0, T2, and T4. PASES has been validated to use in the assessment of young people without chronic disease (factor loadings significant at *P* ​< ​0.05 and ranged from 0.61 to 0.83 for a Caucasian sample) [29]. Items are scored on a 5-point Likert scale ranging from “Strongly disagree” (1) to “Strongly agree” (5), with higher total scores indicating greater self-efficacy. Examples of items include, ‘I can be physically active even if it is very hot or cold outside’, and ‘I have the skills I need to be physically active’. We modified one item of the scale, ‘I can be physically active most days after school’ to, ‘I can be physically active most days after school/university/work’ for relevance to survivors aged 16+ years.

#### 2.6.3 Secondary outcomes

##### 2.6.3.1 Objective physical activity levels

To measure objective physical activity behaviours, survivors will be instructed to wear a research-grade accelerometer (GeneActiv, Activinsights) for five consecutive days (including two weekend days) prior to online exercise consultations with the AEP at T0, T2, and T4. The GeneActiv accelerometer is a waterproof wrist accelerometer that records continuous free-living daily activity and demonstrates good validity and accuracy at both wrist locations (right: *r* ​= ​0.90, left: *r* ​= ​0.91). Survivors will be instructed to return the accelerometer in the paid postage envelope after they have worn the accelerometer for five days. Once the accelerometers are received by the study team, the raw data will be downloaded to a computer, generating one dataset per participant at each time point (i.e., three datasets in total per participant). We will extract the accelerometer binary files from each participant and analyse each dataset based on physical activity intensities, bouts of physical activity, and length of bouts. We will assess the change in objective physical activity levels at T0, T2 and T4 by quantifying daily time spent in various physical activity intensities. Survivors will not be required to wear the GeneActiv and Fitbit activity trackers at the same time.

##### 2.6.3.2 Subjective physical activity levels

To measure self-reported physical activity levels, we will use the modified International Physical Activity Questionnaire for Adolescents (IPAQ-A) [30] administered by the AEP during the telehealth consultations at T0-T4. The IPAQ-A covers questions on four domains of physical activity: school-related physical activity, transportation, housework, and leisure time. It has been validated for use among adolescents and has been used in previous childhood cancer interventions [31].

##### 2.6.3.3 Symptoms of depression

We will use the 12-item Centre for Epidemiological Studies Depression Scale (CES-D-12-NLSCY) [32] to measure survivors' symptoms of depression at T0, T2, and T4. This scale includes 12 items on symptoms of depression such as depressed mood, fatigue, and sleep disturbance with response options, “never or rarely”, “sometimes”, “often”, and “always”. The CES-D-12-NLSCY has good internal reliability (Cronbach *α* ​= ​0.85) when administered to adolescents [32].

##### 2.6.3.4 Readiness to exercise

We will measure survivors' readiness to exercise using the transtheoretical model of behaviour change [33] at T0, T2, and T4. Survivors will be asked to select one of five responses to the following question: “Do you do regular physical activity as described?”. Physical activity is defined as any activity that causes heavier breathing and a faster heartbeat. The expression “regular” physical activity is defined as 4 days or more per week for at least 30 ​min each day. Each of the answer choices corresponds to one of the five stages of the Transtheoretical Model [34]. The word “plan” will be substituted for “intend” based on a previous study in adolescents [35]. The possible answers are: “No, and I do not plan to start doing regular physical activity in the next 6 months” (Pre-Contemplation); “No, but I plan to start doing regular physical activity in the next 6 months” (Contemplation); “No, but I plan to start doing regular physical activity in the next 30 days” (Preparation); “Yes, I have been doing regular physical activity, but for less than 6 months” (Action); or “Yes, I have been doing regular physical activity for more than 6 months” (Maintenance).

##### 2.6.3.5 Perceived enjoyment of exercise

We will measure survivors' perceived enjoyment of physical activity using the Physical Activity Enjoyment Scale (PACES) [36] at T0, T2, and T4. The 16-item scale is scored on a 5-point Likert scale, with higher scores indicating higher level of enjoyment. The PACES has been validated to use in the assessment of children and older adolescents aged up to 18 years [37] and has been commonly used among paediatric oncology populations [38].

##### 2.6.3.6 Health-related quality of life

Health-related quality of life (HRQoL) will be assessed using the EQ-5D-Y-5L [39] at T0, T2, and T4. The EQ-5D-Y-5L has previously been used to assess HRQoL in children diagnosed with cancer [27] and has a good test-retest reliability of 0.84 [40]. The questionnaire comprises five dimensions; ‘mobility’, ‘looking after yourself’, ‘doing usual activities’, ‘pain or discomfort’, and ‘feeling worried, sad or unhappy’, answered on a 5-point Likert scale ranging from (1) ‘no problems’ to (5) ‘extreme problems/cannot’. The EuroQOL Group’s visual analogue scale accompanies the EQ-5D-Y-5L where participants self-report their health on a scale from 0 to 100 (0 ​= ​worst health you can imagine, 100 ​= ​best health you can imagine).

##### 2.6.3.7 Aerobic fitness

We will assess aerobic fitness remotely using the 6-Minute Walk Test (6MWT) during telehealth sessions at T0, T2, and T4. The 6MWT has commonly been used to assess aerobic fitness in childhood cancer survivors [41] and has been validated to be conducted remotely [42]. We have previously assessed aerobic fitness remotely using the 6MWT in a home-setting, administered by parents of survivors [27]. Prior to conducting the fitness assessment, the AEP will screen participants using a short safety screening questionnaire adapted from Bowman et al. [43] (Supplementary Material S1). After passing the safety screening questionnaire, survivors will wear their activity tracker and collect a resting heart rate (after being seated for at least 1 ​min). To complete the 6MWT, survivors will be encouraged to walk as fast as possible (without running) within 6 ​min around a 30 ​m track. We will provide equipment (30 ​m-long rope and cones) for survivors to distinguish the track. Survivors will be instructed to walk around the rope, completing as many laps as possible. A parent or adult will be present to provide verbal encouragement, record the number of laps completed, and record the survivor’s heart rate at each minute. Immediately after the test, the supervising AEP will ask survivors to rate their perceived intensity of the test using the Borg Rating of Perceived Exertion scale (0 ​= ​rest, 10 ​= ​maximal effort) [44] and record their heart rate. The supervising AEP will record each participants' 6MWT distance results and calculate their aerobic fitness percentile level using normative data [45]. We will manually enter their aerobic fitness percentile into our REDCap database.

##### 2.6.3.8 Muscular strength

We will use the five-repetition sit-to-stand test to assess survivors' muscular strength remotely at T0, T2, and T4. The sit-to-stand is a test of strength and endurance relevant to motor skills related to daily activities of living. The five-repetition sit-to-stand test has shown to be a reliable and valid test to measure functional muscular strength in children with cerebral palsy [46], and has commonly been used in paediatric oncology settings [47]. The survivor will be asked to sit up from a chair to a standing position five times with crossed arms as quickly as possible. The test will be timed by their supervising parent or adult, or by the AEP during the telehealth consult. Survivors will be encouraged to use the same chair for each assessment.

### 2.7 Factors that affect implementation to inform scale-up

To explore barriers and facilitators to the implementation and scale-up of Making Moves, we will conduct a process evaluation guided by the implementation framework: the updated Consolidated Framework for Implementation Research (CFIR) [48]. The CFIR aims to predict or explain barriers and facilitators to implementation effectiveness across a range of innovations and settings. We will conduct CFIR-guided semi-structured interviews with potential future implementors/deliverers throughout T0-T3 including healthcare professionals within hospitals or private clinics (e.g., nurses, oncologists, exercise physiologists) or community organisations. The updated CFIR contains 48 constructs and 19 subconstructs arranged over 5 domains (innovation, outer setting, inner setting, individuals, and implementation process) and is a common framework used in healthcare settings to discern future implementation strategies [48]. We will also conduct CFIR-guided semi-structured interviews with survivors and their parents at T2 to explore their barriers and facilitators to using Making Moves to promote survivors' physical activity self-efficacy.

### 2.8 Implementation success of Making Moves

To judge the implementation success of Making Moves, we will collect implementation outcomes including feasibility, acceptability, and cost. We will conduct semi-structured interviews with research staff (e.g., AEP) to seek feedback regarding the implementation potential in their setting, adaptation needs for better fit in their context, and potential implementation strategies needed to support uptake. We will also record implementation data throughout the intervention period and at T2. This implementation data will include healthcare professional time, resources used, and any costs to implementation for survivors and parents.

#### 2.8.1 Feasibility

Feasibility according to implementation outcomes defined by Proctor et al., is the extent to which a new treatment or innovation can be successfully used or carried out within a given setting [49]. We will assess feasibility as a potential explanation of the success of Making Moves, reflected in ≥70 ​% recruitment rate, ≥70 ​% retention rate, ≥70 ​% participation rates (number of modules completed and AEP telehealth sessions attended) and no adverse events (defined as an unfavourable or unintended sign, symptom, or diseases associated with study participation). We will grade each adverse event using the National Cancer Institute’s Common Terminology Criteria for Adverse Events [50]. We will assess adverse events via an email message prompt with a link to a REDCap questionnaire to survivors aged 18+ years and to parents of survivors who are <18 years of age. Text messages will be sent to survivors/parents every two weeks during the intervention (i.e., at weeks 2, 4, 6 and 8).

#### 2.8.2 Acceptability

Acceptability is defined as the perception among implementation stakeholders that a given innovation is agreeable, palatable, or satisfactory [49]. We will assess the acceptability of Making Moves from survivors (including the intervention, digital aspect, educational modules, and AEP services) at T2. We will use an adapted version of the Youth Satisfaction Questionnaire [51] and use open-ended questions regarding participants’ most enjoyed and least enjoyed features of the program and any suggestions for improvement. We will also use a 2-item benefit and burden questionnaire reported on a 5-point Likert scale (0 ​= ​not at all, 4 ​= ​very much), “Was participation in this study beneficial to you in any way?” and “Was participation in this study burdensome for you in any way?”. To assess the acceptability of the module content, survivors will rate their satisfaction at the end of each module using a 5-point star scale (1 star ​= ​very dissatisfied, 5 stars ​= ​very satisfied).

#### 2.8.3 Cost

Cost is defined as the cost impact of an implementation effort [49]. We will identify resources relating to the intervention and value any costs associated in delivering Making Moves, collect costs related to the operation, delivery, and uptake of Making Moves, and collect costs related to the intervention incurred for survivors and parents. To evaluate costs incurred to survivors, parents will complete a study-specific questionnaire at post-intervention. The study coordinator and AEP involved in delivering the intervention will also keep detailed notes throughout the intervention on the intervention costs, and any barriers and facilitators to the delivery of the program, and potential improvements or strategies that may be important for future implementors.

### 2.9 Sample size

A sample of at least 53 participants will provide greater than 80 ​% power to detect a difference in the mean physical activity self-efficacy score after participating in the Making Moves program. We define a meaningful change in physical activity self-efficacy as one that is a standardised increase of 0.4, informed by previous studies assessing physical activity self-efficacy in adolescents [52,53]. With 53 participants, if the within-individual correlation in the outcome between baseline and post-intervention is 0.5 or greater, a paired t-test would have at least 80 ​% power to detect a meaningful difference in mean scores between time-points, using a two-sided significance level of 5 ​%. To allow for 30 ​% attrition from pre-to post-intervention (based on our pilot), we aim to initially recruit at least 76 participants for the baseline assessment.

### 2.10 Analysis

To examine the potential effectiveness of the intervention on the primary outcome (physical activity self-efficacy), we will calculate the change in self-efficacy scores from T0 to T2 (primary endpoint) and from T0 to T4 using mixed effects regression models, with random intercepts per individual, and fixed effects for the time points. We will perform similar analyses for secondary outcomes, modified to suit the outcome measure. To report on participant characteristics, we will use descriptive statistics as appropriate. Semi-structured interviews will be recorded and transcribed using Microsoft Teams, and verified for accuracy. We will analyse interview data deductively and thematically according to the CFIR determinants (intervention characteristics, outer setting, inner setting, process, and characteristics of individuals) and the TDF domains (e.g., knowledge, skills, beliefs about capabilities, beliefs about consequences, motivation, and goals etc.).

### 2.11 Ethics and dissemination

This research study is approved by the Sydney Children’s Hospital Network Ethics Committee (2023/ETH01614) and is registered on the Australian New Zealand Clinical Trials Registry (ANZCTR12623000188639). Our team will disseminate information regarding this research study via quarterly newsletters sent to our collaborators and partners (e.g., our Youth Advisory Board, The Kids' Cancer Project). Quarterly newsletters will include study updates on recruitment and preliminary feedback from participants or study staff. Young survivors involved in our Youth Advisory Board have contributed to the conception and development of this intervention and will be involved on an ongoing basis. Our team also plans to submit abstracts to medical and scientific conferences and publish manuscripts in peer-reviewed journals.

## 3 Discussion

This study has been designed to assess the potential effectiveness of an online physical activity program, called ‘Making Moves’ on childhood cancer survivors' physical activity self-efficacy. We previously piloted a previous iteration of this distance-delivered program and found that it was feasible to deliver in a home-setting and acceptable among young survivors [54]. We subsequently established a Youth Advisory Board and underwent an iterative process with consumers to improve the program by addressing survivor and parent feedback from the pilot study. Additionally, we conducted multi-perspective focus groups and interviews with survivors, parents, and healthcare professionals to explore their experiences and priorities of using digital health to support healthy behaviours. Findings from these focus groups and collaborations with our Youth Advisory Board led to the co-development of Making Moves. A key strength of our intervention is the person-based co-design approach that we adopted in the development and evaluation cycles when making improvements based on data gained during evaluation phases [55]. Working with consumers in the design, development, decision-making, and evaluation of the intervention is essential to understanding their perspectives and ensuring the intervention is engaging, relevant, and sustainable [56]. Moreover, our multi-perspective approach is highly valuable as it captures differing views based on the experiences of healthcare professionals and parents that are imperative to consider when delivering interventions for young survivors.

This type I hybrid effectiveness-implementation trial also seeks to determine the potential for future adoption, implementation, and sustainability in real-world settings. Digital health interventions are a promising solution to reach and engage young people diagnosed with cancer, including those living in regional and remote areas [57]. Preliminary results have shown that distance-delivered interventions are feasible and acceptable, and may improve health behaviours such as physical activity levels [18,19]. Factors critical for successful integration and implementation within typical clinical settings are also yet to be determined. Our proposed study aims to improve understanding of factors that may affect future adoption and implementation decisions and explore potential avenues for integrating Making Moves into standard practice. Our process evaluation will enable us to identify strategies and barriers related to recruitment, retention, and implementation that we may not otherwise learn if not for this hybrid study design (i.e., conducting an effectiveness trial then subsequently implementation research). In addition, our analysis of the program costs will provide an important insight into the requirements of program delivery and uptake and may assist with considerations for future stakeholders seeking to implement this program. Combining these strategies will allow us to identify what is needed to support implementation in the real world and identify the barriers and facilitators to implementation that will inform our future selection of appropriate implementation strategies. However, a limitation of this trial is its single group design, which means the trial will not compare survivors who participated in the intervention to a usual care group or the intervention without the AEP sessions. This type I trial will be essential for gathering baseline data, priming the study for the next phase where we will conduct comparative analyses using implementation strategies identified from this study.

This study will help to provide evidence on the potential effectiveness of Making Moves in improving childhood cancer survivors’ physical activity self-efficacy, while allowing us to better understand the implementation context in several clinical settings. Our distance-based delivery strategy will bridge the barrier of distance by reaching survivors and families who may struggle to access specialised exercise support in cancer survivorship [58]. Findings from this study will continue to advance understanding of the critical role of exercise in cancer care [1], which is not yet widely implemented within paediatric oncology. Ultimately, our study will help to equip young survivors with the resources to engage in physical activity to improve their self-efficacy, health outcomes, and quality of life after cancer.

## Author contributions

LH and CW conceived the study. LH, CW, CS, YAB, RL, RC, NT, CS and YAB contributed to the design of the study. LH drafted the protocol with input from all authors. MWD provided statistical expertise. LH, JJ, KJ, DD, and RC will contribute to the recruitment of participants. All authors critically reviewed the manuscript.

## Data statement

Data available within the article or its supplementary materials.

## Funding

This work is supported by The Kids' Cancer Project. Dr Lauren Ha and Dr David Mizrahi is funded by The Kids' Cancer Project. Dr Christina Signorelli is supported by a Cancer Institute NSW Early Career Fellowship (2020/ECF1144). Prof Claire Wakefield is supported by an NHMRC Investigator Grant (APP2008300).

## Declaration of competing interest

The authors declare that they have no known competing financial interests or personal relationships that could have appeared to influence the work reported in this paper.
