# Supplementary material for: ‘Making Moves’: Protocol for a hybrid effectiveness-implementation pre-post trial of a co-designed online physical activity program for childhood cancer survivors
Source: JSAMS Plus. 2025 Apr 12;5:100099. doi: 10.1016/j.jsampl.2025.100099 (PMC13008450; doi:10.1016/j.jsampl.2025.100099)
Supplement: Multimedia component 1 [file mmc1.docx]

# **Supplementary Materials**

## **Supplementary Material S1 – Physical Activity Safety Screening Questionnaire**

Please answer the following safety questions before starting the physical activity assessments. If you answer 'no' to any of the below DO NOT begin the physical activity assessments. Please ensure that you can confidently answer 'yes' to all of the questions below before commencing the physical activity assessments to avoid any risk to your health.

| **Physical Activity Safety Screening Questions** | **Yes** | **No** |
| --- | --- | --- |
| 1. Do you have an adult at home who can supervise you whilst completing this test? |  |  |
| 1. Are you wearing sturdy shoes – that don’t slip and have support at the heel? |  |  |
| 1. Have you had a recent injury or fall that would impact your ability to complete the test today? |  |  |
| 1. Are you feeling dizzy, cool, or sweaty? |  |  |
